# A Case Report of Home-Based Cognitive-Behavioural Treatment for Late-Onset Post-Traumatic Stress Disorder, Triggered by Mask-Wearing in the Context of the COVID-19 Pandemic

**DOI:** 10.1177/15346501221102915

**Published:** 2022-12

**Authors:** Adham Hanafi

**Affiliations:** 1 1555 University of Bath and NHS Somerset Foundation Trust, UK

**Keywords:** post-traumatic stress disorder, delayed-onset post-traumatic stress disorder, late-onset post-traumatic stress disorder, CBT, cognitive model of post-traumatic stress disorder, COVID-19, physical health, home-based therapy

## Abstract

A small but clinically significant number of people experience delayed-onset Post-traumatic stress disorder (PTSD); symptoms of trauma years after the events which are now being re-experienced. The following case report describes the use of the cognitive-behavioural treatment for PTSD with a woman experiencing flashbacks to domestic abuse endured more than 20 years ago. Mask-wearing mandated as a result of the COVID-19 pandemic triggered non-contextualised memories of life-threatening physical violence by an abuser who covered his face. She had been managing her flashbacks and intrusive thoughts with both behavioural and experiential forms of avoidance. An 18-session intervention was provided in her own home due to physical health difficulties. Treatment focused on managing hyper-arousal, reducing thought suppression, in-vivo exposure, stimulus-discrimination and re-contextualising traumatic memories. Regular outcome measurements were kept and results are presented as a single-case experimental design in ‘AB’ format (i.e. baseline period pre intervention). Symptoms of trauma fell to levels non-indicative of PTSD and speak to the evidence base for this modality, even when applied to delayed-onset difficulties in a non-traditional therapy setting. This conclusion is lent extra credence by an experimental design with good internal validity.

## 1 Theoretical and Research Basis for Treatment

Post-traumatic stress disorder (PTSD) can follow the exposure to death (or the threat of it), serious physical harm and sexual violence. The DSM-5 diagnostic criteria include the presence of symptoms such as intrusive memories of the event(s), nightmares, dissociative reactions (e.g. flashbacks) and psychological distress resulting from exposure to internal or external reminders of the trauma (accompanied by strong physiological reactions). These must persist beyond the traumatic event for longer than 1 month (American Psychological Association, 2013). Other key criteria include avoidance of reminders of the event(s), which may be internal (e.g. thoughts and feelings) or external (e.g. people or places). A negative impact on thought processes about oneself, other people or the world is also common. This is often attendant with a range of negative emotion such as fear and guilt. Irritability and hyper-vigilance (being constantly ‘on guard’ for potential threats) are also symptoms of PTSD (APA, 2013). The disorder impairs social and occupational functioning and is frequently accompanied by difficulties with depression and anxiety (Flory & Yehuda, 2015).

A 2014 UK-wide psychiatric survey estimated prevalence of PTSD at 3.7% of men and 5.1% of women (NICE, 2018). However, among certain groups rates are higher, for example the military and police (Dyball et al., 2019).

Statistics on the prevalence of PTSD vary but hover around 4.5% (MIND, 2017). However, they suffer from a host of confounding factors, not least treating varied groups homogeneously. More assuredly, it can be said that the prognosis of PTSD is poor, impacting in deleterious ways on both cognitive functioning and physical health.

The link between trauma and physical health difficulties is clear. A 2018 study found that among over 3000 military veterans, PTSD was associated with increased odds of respiratory disorders such as asthma and COPD (significant odds ratio compared to sub-clinical controls, OR 2.15, 95% CI 1.54–3.01; El-Gabalawy et al., 2018). The same study found increased odds for a range of other physical health problems for those (of any age) with PTSD including heart disease (OR 0.93, 95% CI 0.72–1.38) and arthritis (OR 1.04, 95% CI 0.79–1.36).

While many theories have tried to take account of PTSD symptomology, this overview focuses on the cognitive model as it forms the basis of its treatment using CBT.

### The Cognitive Model (Ehlers & Clark, 2000)

The cognitive model suggests that PTSD develops when past life-threatening events, or their consequences, are appraised as a threat to current safety. The appraisals might be those made during the event itself (e.g. ‘I’m going to die’), the way in which one coped (‘I’m vulnerable now’) or symptoms of the disorder itself (‘I’m going mad’).

The cognitive model leans on the earlier Dual Representation Theory (DRT; Brewin et al., 1996) which outlined two types of memory formed during trauma. The first, verbally accessible memories, are laid down in the hippocampus and constitute what the person was able to attend to consciously before, during and after the event. Given this, they are retrievable ‘on request’. However, during traumatic events, amygdala activation stimulates the autonomic nervous system at the expense of hippocampal activity (Shin, 2006). This means that some information, particularly sensory experience, is stored unconsciously as Situationally Accessible Memories (SAMs). Situationally accessible memories are used to explain flashbacks as a traumatic memory being triggered by situationally available stimuli. The cognitive model goes further than DRT by placing emphasis on the cognitive and behavioural strategies that maintain the disorder. Behaviourally, these include hyper-vigilance (Smith et al., 2009) while cognitive strategies include thought suppression (the conscious and ineffectual attempt to try and not think distressing thoughts; Wegner, 1994).

Empirically, some aspects of the model are better supported than others. Controlling for specific event factors that may impact severity is hard to achieve experimentally. Nonetheless, aspects of the model shown to have particularly strong support for predicting PTSD are mental defeat, negative appraisals of the trauma symptoms and use of avoidance to cope (Brewin & Holmes, 2003).

### Treatment

The cognitive model gives rise to three treatment implications. Firstly, the importance of better contextualising the trauma memory in time and place to reduce flashbacks. Secondly, restructuring negative appraisals of the trauma or its impact/symptoms functioning as psychological threats. Thirdly, reducing avoidant behavioural strategies and facilitate sustainable coping by challenging anxiety-laden predictions about re-contact with trauma-related stimuli. NICE guidelines recommend several forms of treatment to achieve these aims including cognitive, prolonged exposure and narrative exposure therapies (all fall under a broad ‘CBT-Trauma’ label), as well as EMDR (NICE, 2018).

At a broad level, research into CBT’s efficacy in treatment of PTSD has consistently shown it to be more effective than wait-list controls (Watkins, Rothbaum & Sprang, 2018). Overall, it has more empirical support comparative to other psychotherapies (Weber et al., 2021). Identifying the ‘active ingredients’ under the CBT-PTSD umbrella, Acierno et al. (2016) carried out a randomised controlled trial (*n* = 184), containing an exposure to trauma-related stimuli condition with military veterans. Importantly for the present study, they compared the efficacy of (in vivo and imaginal) exposure-based treatment both at home (via telephone therapy) and face to face in a clinic, finding no inferiority (and almost equal efficacy) for the home-based intervention (a mean difference of −0.66 on the PTSD checklist, PCL, at 12 month follow-up).

Langkaas et al. (2017) looked at the comparative efficacy of two CBT-based interventions, prolonged exposure and imagery re-scripting, both of which assume slightly different underlying maintenance structures for PTSD. The study, undertaken at a Norwegian (non-psychosis) inpatient clinic, found both to be effective at reducing symptomology following a 10 session intervention, sustained at 12 month follow-up. There was no significant difference in the utility of treatment condition (*n* = 71). This was notable as prolonged exposure has been suggested as less efficacious when fear is not the primary negative emotional component of a person’s distress. However, exposure in this study alleviated trauma symptoms even when anger and shame were the predominant affective experience.

The manualisation and adaptability of CBT – in its broadest sense as a ‘network’ of theoretically connected interventions – is in part responsible for its growth and application to a wide-range of mental health difficulties in a variety of settings (Pickersgill, 2019). One of the most significant adaptations has been expansion into home-based treatment. This most commonly looks like digital therapy using online software monitored by a trained professional, or telephone therapy, but can also include face to face sessions in a client’s home. This is increasingly true for older adults who may not be able to access therapy in traditional settings due to physical health or other accessibility difficulties, for example, use of transport. However, while the efficacy of digital and telephone therapies continues to receive burgeoning empirical attention, the research literature for home-based, in-person, CBT (with any client-group) is small and, by extension, even more so for investigating disorder-specific utility. Nonetheless, a 2017 RCT (*n* = 134) investigated home-based, face to face CBT with older people living in the rural United States. A range of trans-diagnostic cognitive-behavioural techniques were used (e.g. thought diary; spotting cognitive distortions) and found improvements in generalised anxiety and phobia-specific anxiety, compared to a minimal input control condition. By the end of the 16 session intervention, generalised anxiety levels reduced significantly in the treatment group (F (1,101) = 4.97, *p* = .03). Phobic anxiety scores also improved significantly comparative to controls (F (1,102) = 4.52, *p* = .04) (DiNapoli, Pierpaoli, Shah, Yang and Scogin, 2017).

### Delayed-Onset PTSD

Delayed-onset PTSD is important to comment on given the case report to follow. It refers to the small but clinically significant number of people who develop symptoms long after the traumatic event(s) took place. Understandings of why this happens are limited but one explanation is that SAMs remain dormant without a given stimuli for the memories (Ehlers & Clark, 2000). This is of particular relevance to people of middle or older age, who, inherently, have had more time to be re-exposed to reminders of trauma. An alternative hypothesis is that later events change the meaning of earlier ones (e.g. new evidence meaning a historic sexual assault is appraised later-on as having been exactly that). And a third idea is that people may have always displayed some symptoms, though not of a clinical level, and additional life stressors such as bereavement exacerbate these (Kuwert et al., 2013); notwithstanding the impact on cognitive and physical functioning that may have already taken place.

## 2 Case Introduction

The following report employs a single-case experimental design in ‘AB’ format; PTSD and co-morbid symptomology was established during a 3-week baseline phase (phase ‘A’) prior to commencing an 18 session intervention (phase ‘B’).

DH (an initialled pseudonym), in her late 50s, was referred for psychological therapy in an NHS Community Mental Health Team. The brief referral information indicated the main problem as a ‘phobia of masks’; the face coverings that had become mandatory in many public places as a consequence of the COVID-19 pandemic. DH requested that sessions were carried out at her home due to mobility problems and fear of seeing masks.

## 3 Presenting Complaints

On first meeting, DH was essentially agoraphobic, stating that she felt too fearful to go outside as she would see people wearing masks. Masks trigged intrusive thoughts about imminent threat, a commanding ‘voice’ telling her to escape, and strong autonomic arousal. No formal diagnosis had been made but DH was taking prescribed SSRIs and understood these to be for her depression (the loss of her son meant ‘I’ll always be depressed’).

DH suffered with COPD, arthritis and chronic lower back pain. These significantly reduced her mobility and desire to go outside.

## 4 History

DH was white and lived in a deprived area of a city in the south-west of England. She attended school until the age of 16 and married in her early 20s. She had three children over the next 6 years. During this marriage, from the late 1980s to the late 1990s, she suffered domestic violence and other forms of psychological abuse. A very serious assault precipitated the end of the relationship. She was rehoused by the city council’s housing department, with her children, and a period of employment as a dinner lady followed which lasted until the early 2000s. DH’s eldest son committed suicide in 2007. She managed her grief using alcohol and experienced difficulties with severe depression, symptoms of which continued to the present day. Her youngest son was in prison for a fire-arms related offence. DH lived with her daughter who was in her mid-30s and provided a range of support, for example, shopping and pet-care.

## 5 Assessment

Assessment took the form of semi-structured interview over three 60 minute sessions. Masks were threatening to DH as she believed that she needed to read people’s facial expressions in order to be safe. While the need to ‘suss’ people out predated her marriage, DH learned to look at her husband’s face when he returned home from work or the pub in order to discern whether she would be beaten-up that evening. Moreover, she came to associate a covered face with certain physical harm. DH did not have a strong sense of why he hurt her while covered (e.g. ‘because he was mental’).

She described one critical incident in which he pinned her on the living room floor, her hands under his knees, holding an ice pick above his head and saying he was going to kill her. At this point, police burst in, having been called to the disturbance by a neighbour. During this event, he was wearing a face-covering that only showed his eyes (perhaps a balaclava, DH was not sure). Following this, DH’s mother moved her and her children back in to the family home and she was eventually rehoused.

The belief in the need to see people’s faces to evaluate her own safety, and her self-assessment as being very good at this, was longstanding. While this was easy to understand as a consequence of the physical and psychological abuse endured, her nearly 60 years in a socio-economically deprived area were assessed as both vulnerability and maintaining factors for her difficulties. Her exposure to violence (stories of violent siblings and her own episodes of physical aggression) and crime (son in prison) spoke to a history in which the development of a hyper-vigilant behavioural strategy would have been highly adaptive.

While the basic referral information had suggested DH’s problem was a phobia of masks, this was not evidenced by the more detailed information elicited during assessment. Rather, DH’s presentation suggested that masks were triggering flashbacks of domestic abuse. For example, a dissociative process would be triggered by the practioner’s use of the word ‘mask(s)’ alone; she would withdraw, pressing her chin down against her neck, visibly perspiring and unable to hear questions asked of her for several minutes.

The historical severity of domestic abuse, in particular one incident in which she believed she was going to die, flashbacks to this event with palpable ‘nowness’, intrusive thoughts, in-session dissociation, hyper-arousal and hyper-vigilant safety strategies all spoke to post-traumatic symptomology. Given this, the post-traumatic stress checklist (PCL-5) was used to specify prevalence. The PCL-5 is a 20 question, self-report, measure on a likert scale from 0 (not at all) to 4 (extremely). A sample item is ‘Over the past month, how much have you been bothered by being “super-alert”, watchful, or on guard?’ It has a maximum score of 80 and its four sub-scales mirror DSM-5 symptom clusters; hyper-arousal, avoidance, intrusions and mood/cognition. The PCL-5 has demonstrated strong reliability (α 0.96; Bovin et al., 2016). While the measure is not diagnostic, DH scored 55/80, suggesting ‘probable PTSD’ (≥33; Weathers et al., 2013).

Due to frequent comorbidity with PTSD, the GAD-7 and PHQ-9 were also administered during assessment. The GAD-7 is a 7 item self-report measure used to assess the severity of anxiety symptoms (Spitzer, Kroenke, Williams & Löwe). On a likert scale from 0 (not at all) to 3 (every day), it has a maximum score of 21, demonstrating strong reliability (α 0.92; Zhong et al., 2015). DH scored 18 on this measure which fell within the ‘severe’ anxiety range (Spitzer et al., 2006).

The PHQ-9 is a self-report measure of depression symptoms. Like the GAD-7, it is not diagnostic but provides an indication of severity. It has 9 items on a likert scale from 0 (not at all) to 3 (every day), with a maximum score of 27 and evidence of strong reliability (α = 0.86; Kroenke et al., 2001). DH scored 19, falling into the ‘moderately-severe’ range.

### Client Goals

Assessment identified the following goals:

• In 2 months’ time, walk my smaller dog for short-periods of time with my daughter
• In 3 months’ time, go food shopping with my daughter at a large supermarket
• In 5 months’ time, go on a week-long holiday with family to a caravan park

DH rated herself as 0/10 on an idiosyncratic measure of current capability to carry out these activities tomorrow.

## 6 Case Conceptualization

Masks were triggering flashbacks to a traumatic incident of domestic abuse 20 years ago. Episodes of re-experiencing had a clear sense of ‘nowness’, with DH saying that during them ‘it’s as if he’s right back over me with the ice pick’. Masks were a previously unavailable stimulus for poorly elaborated and non-contextualised memories.

While DH could readily identify the certainty of death in her thoughts (‘he told me he was going to kill me and he would have done it’), it was the consequence of this outcome on her children that was most traumatic. DH’s self-image was strongly that of a mother, a protector and a custodian of others (including her many animals). Dying as a result of domestic abuse would have precluded her from enacting these values. While a survivor narrative may have been appropriate for the escape from domestic violence, the tragic suicide of her eldest son in the early 2000s served as a barrier to internalising any such story.

Her son’s suicide had a profound effect on DH, not least in a belief that she would be better off dead, ‘to be with him again’. DH had no intentions to end her life, her two other children being protective factors but, nonetheless, was an important part of the formulation because the loss contributed to a view of the world as unjust, unpredictable, and of which she wanted little part. DH was frequently angry and irritable in her attitudes toward others (e.g. those who wore masks) and told stories of physical aggression prior to her current difficulties. Her suicidality functioned to reassure her that she would 1-day reunite with her son, speaking, on top of her ‘generalised’ anger toward others, to a complex and unresolved grieving process. Her belief that this loss would leave her depressed in perpetuity further evidenced a key facet of a her psychological functioning, that is, the concreteness of thought that led her to be convinced of her abilities to mind-read other people’s mal-intentions toward her, particularly if they wore a face covering.

When triggered by masks, DH described a threatening male ‘voice’ present in her mind. The voice would shout that mask-wearers were ‘going to get me’ and she was visibly distressed when this took place in-session. DH said that versions of this voice had been with her throughout her life, which helped rule out other understandings (e.g. internalised voice of her abuser). It was formulated as a form of intrusive thought whose function was to alert DH to potential danger. DH managed intrusions by telling them to ‘fuck off’ and this was identified as a form of thought suppression. She was actively trying to control (get rid of) an unwanted cognitive experience by attempting not to think about it. Her lack of success with this strategy (the voice got louder and she became hyper-aroused as she attempted in vain to dismiss its warnings about imminent danger), also spoke to this conceptualisation (Wegner, 1994).

DH employed a range of behavioural strategies to prevent re-experiencing. The starkest was to have stopped going outside, even to the garden. DH’s agoraphobia was hypothesised to be maintaining symptoms via three key mechanisms; (1) building reliance on ineffectual coping strategies (e.g. thought suppression), (2) reinforcing negative appraisals of the self (‘I can’t cope with masks’), others (‘people will hurt me if I can’t read them’) and the world (by mimicking the level of control she was under during marriage) and (3) preventing the trauma memory from fuller integration into autobiographical memory (Ehlers & Clark, 2000).

## 7 Course of Treatment and Assessment of Progress

On-going assessment of progress was carried out using the measures described in the Assessment section above; the PCL-5 (administered monthly), PHQ-9 (weekly) and GAD-7 (weekly).

The PHQ-9 and GAD-7 were used to establish a baseline level of functioning. This comprised measurement at three time-points during the assessment phase. Given that the hypothesis of PTSD did not emerge until later in the assessment period (as dissociative processes in response to semi-structured interviewing questions were observed), earlier measurement using the PCL-5 during baseline did not take place (see Limitations). Unfortunately, due to health service constraints, it was not possible to achieve a baseline phase that did not co-vary with the assessment period.

An idiosyncratic measure was also created; ‘how capable are you of going to the supermarket tomorrow with your daughter?’ DH would give a response on a scale from 0 to 10 (zero being completely unable and 10 being fully able). This was taken at the beginning, middle and end of therapy and progress would also be measured against DH’s goals.

### Treatment Hypotheses

The first hypothesis was that DH was engaging in a form of thought suppression by telling thoughts to ‘fuck off’. Cognitive restructuring was planned to shift the meaning of this ‘voice’ by learning to see it as serving an important, (albeit over-) protective, function. Efficacy would be measured with the GAD-7 and PCL-5.

The second hypothesis was that avoidance of masks reinforced the beliefs that she needed to see faces to be safe and that masks were dangerous. This fuelled hyper-vigilance resulting in the noticing of more threats. Behavioural experiments were planned to show that masks were safe. This would also function as in-vivo exposure to reminders of the trauma and be measured with the PHQ-9 and PCL-5.

A third hypothesis was that memories of the index traumatic event were poorly integrated into autobiographical memory, likely due to the high levels of autonomic arousal during the life-threatening physical abuse. Elaborating these using stimulus-discrimination and reliving interventions were planned to place memories in their time-limited context. Efficacy would be measured using the PCL-5.

### Session by Session Intervention Outline

**Figure 1.**
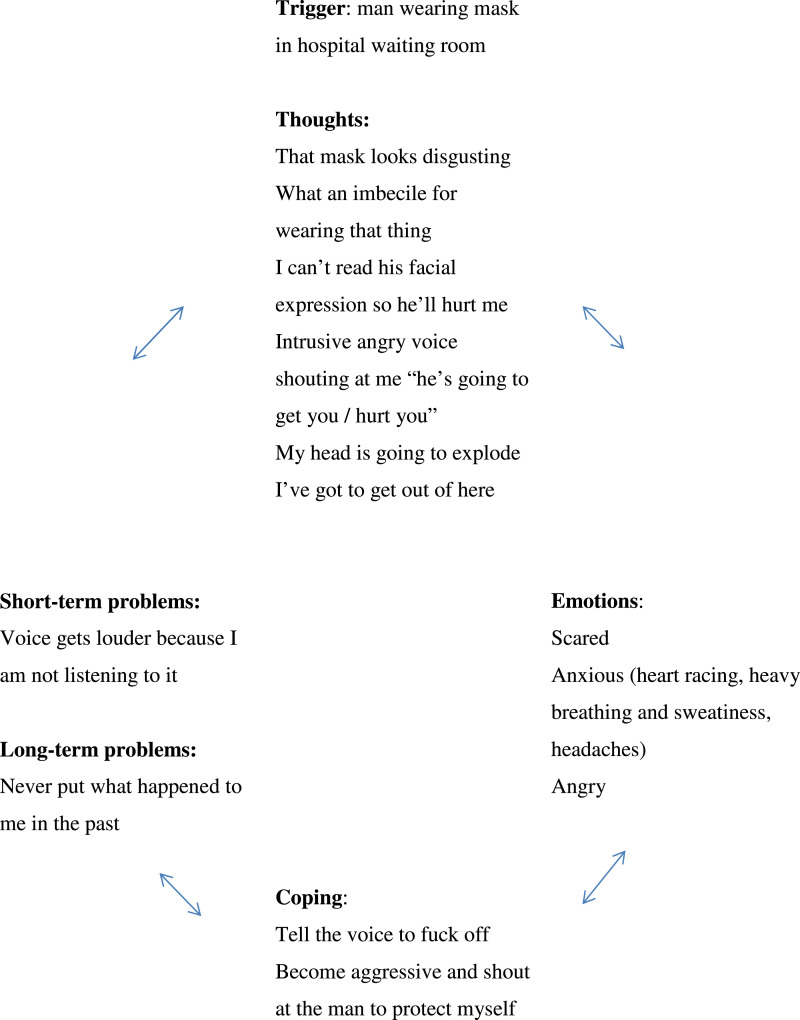
Shared cross-sectional formulation of flashbacks.

After the assessment period, the fourth session included collaborative, cross-sectional, formulation of a given flashback (Figure 1). This helped DH to identify her own cognitive response to masks. She understood that her threat-based predictions of the harm mask-wearers posed elevated arousal levels. This session also introduced key stabilisation techniques. These were vital to develop early on since DH’s dissociative states in response to the word ‘mask(s)’ would take extended time for her to down-regulate. The main technique used was a truncated form of grounding; encouraging DH to name three things she could see, two she could hear and one she could touch. The brevity of this enhanced its adoption as treatment moved into its fifth session, where metaphors describing the experience of trauma symptoms (e.g. ‘the memory’ as an over-filled cupboard in need of looking at and reorganising) were used to provide the rationale for both stabilisation techniques and the interventions to come. Further, these were important psycho-educative elements aimed at helping DH make sense of her symptoms, in particular re-experiencing, disabusing her of the notion that she was ‘going mad’.

In session six, the ‘pink elephant’ exercise demonstrated the consequences of ‘trying to ignore or get rid of the voice by telling it to go away’. DH learned that her current coping strategy had the unintended consequence of making the voice, on sight or sound of mask-related stimuli, louder. Socratic dialogue (Padesky, 1993), particularly exploring any positives to the voice, was a fruitful line of enquiry. DH was able to identify the voice’s protective function. This led to an update of the formulation and treatment was subsequently positioned as attempting to turn the voice ‘down but not off’. This was achieved through questioning processes aimed at reducing thought suppression and restructuring appraisals of the voice as a sign of impending attack; for example, ‘What would happen if you said “thanks, I know you’re trying to protect me, but I don’t need you quite so loud right now?”’

Session 7 targeted hyper-vigilance by using a behavioural experiment described on the OxCADAT website (PTSD Therapy Materials, 2022) to proactively ‘find threats’ in the room. The intention here was to demonstrate that searching for threats finds them, and link this to her habitual attempts to ‘suss’ people out, which were currently blocked by mask-wearing and leading to intensified predictions of harm.

Sessions 8, 9 and 10 focussed on stimulus-discrimination (see Appendix A for example worksheet), a cognitive tool designed to get DH verbalising (and therefore better contextualising) the traumatic event in greater detail (at this stage in the past tense). The worksheet assisted with recognition of the ways in which her current life contradicted the predictions she made at the time of the attack. It was of particular importance that emphasis was placed on the fact that she *and* her children had survived this event; she had continued to raise them long after the abuse was over. This was because loss of her children (to her abuser) was the worst predicted consequence during the moments she believed she was about to die.

Sessions 11 and 12 targeted beliefs about masks being a sign of impending threat using in-vivo exposure tasks. These were set up as behavioural experiments, an example of which is provided in Table 1. In-vivo tasks challenged some of DH’s more fantastical thinking around the threats that masks posed, such as the potential for them to move of their own accord. They furthered understanding that she did not need to see faces in order to ‘suss’ people and remain safe. DH learned that avoidance of masks prevented her from developing skills to live with them. As she enjoyed watching the many sports tournaments taking place during the summer of 2021, between-session tasks lent on this by simply encouraging DH not to change channel when crowd members wearing masks were displayed on the TV screen. She could further disprove her negative predictions about contact with masks or continue stabilisation skills should she in fact be trigged by images.

**Table 1.** Typical Behavioural Experiment Completed by DH to Test Harmfulness of Masks.

Situation	Experiment	Prediction	Likelihood/evidence	Outcome
Masks prevent me from seeing faces which means I cannot tell if I am safe or not. I avoid all masks	Therapist to put mask on during session	Therapist will come really close to me despite saying he will not Therapist may attack me once he has mask on Mask will move of its own accord and come toward me	Therapist breaking promise to stay in seat Therapist hurting me Mask moving off therapist’s face	Mask did not move nor did therapist hurt me. However, that is because you work for the NHS and I know you are a good person. Other people are different. It was still really horrible having one being worn in my house as I have banned them Intrusive thoughts were saying you were going to attack me which was really scary

Session 13 (but in actuality a thread throughout treatment) focused on re-capping responses to intrusive thoughts. Essentially, encouragement to engage with what the voice was orientating her towards, rather than trying to suppress it. DH began to display more confident responses such as ‘I’m safe, they are [mask-wearers] not going to hurt me’, which proved effective in maintaining low levels of autonomic arousal. This was built-on by starting to accompany her daughter on trips to a large local supermarket, full of shoppers wearing masks, experiencing significant anxiety and deploying safety behaviours (e.g. trying to remain in-front of mask-wearers), but no flashbacks and able to resist the desire to flee.

Given this progress, sessions 14 to 17 were spent re-living the trauma memory and updating hotspots, elaborating and contextualising the trauma memory. DH initially struggled to stay in the present tense while describing the events. However, key hotspots caused her to flinch during the re-telling. Detail of these hotspots was elicited and noted by the practioner. DH was then guided through reliving while updating the key hotspots predicting death (‘I did not die’) and hearing her children crying in the next room and fearing for their safety (‘we all escaped, survived, and I continued to be a mother to them’).

Session 18 was spent creating a relapse prevention plan. This included a detailed list of the safety statements she had developed during cognitive restructuring and re-living. However, in both a sign of progress made and work still to be done, DH went on holiday just prior to this session (as per her goals). She disclosed a significant episode of re-experiencing during the trip in which she had been sitting outside of a café, next to a take-away restaurant, with a long queue of people wearing masks spilling out onto the pavement. The proximity and amount of masks were the key variables DH identified as most distressing; triggering intrusive thoughts and building to flashbacks of the critical incident once again. Crucially, however, she had remained at the table rather than escaping, making attempts to regulate the distress by engaging with the threat-based intrusions; ‘I just said to “him” that we were safe and no-one was going to get us’, ‘I knew if I ran off it would have sent me right back to the beginning of all this’. This demonstrated an understanding of the role of avoidance but also implementation of a central strategy to reduce thought suppression.

### Results

**Figure 2.**
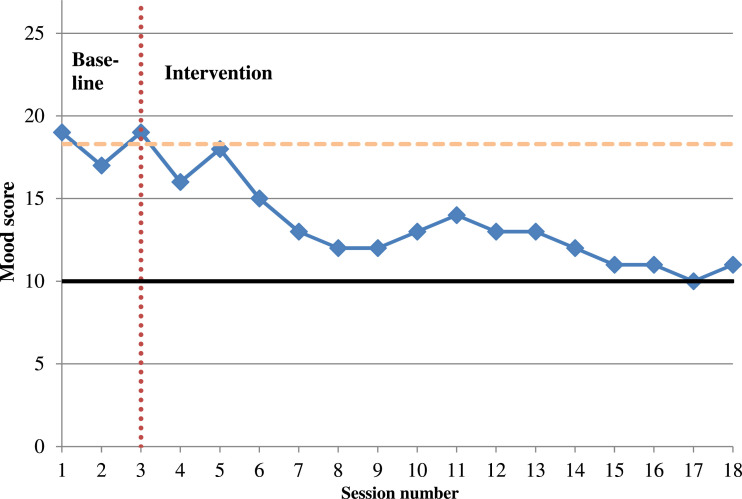
Line graph showing DH’s depression score as measured by the PHQ-9. *Note*. The solid black line represents the start of the ‘moderate’ depressive symptoms range (≥10). The horizontal orange dashed line indicates the mean score of symptoms for the baseline period. The vertical red-dotted line marks the end of baseline period and commencement of intervention.

Symptoms of low mood were measured using the PHQ-9 at three time-points during the assessment phase, to establish a baseline score (mean 18.3). At the end of treatment, a score of 11 indicated that clinically significant change had not taken place (≤10). However, a difference of greater than six between baseline period and end of therapy indicated that reliable change had taken place (IAPT, 2014). Figure 2 illustrates these results.

**Figure 3.**
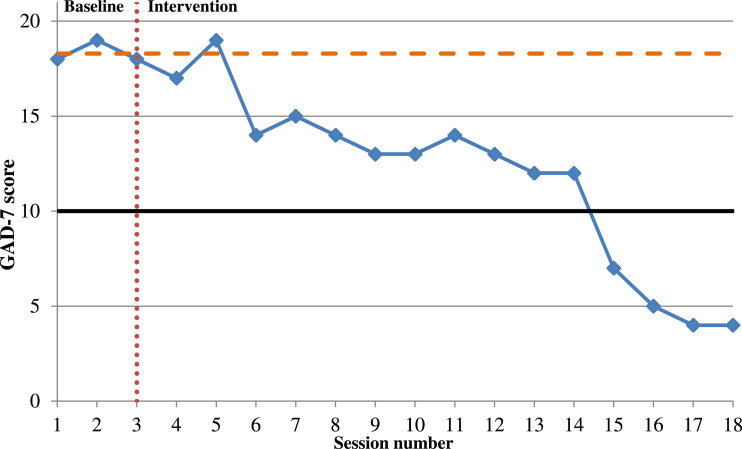
Line graph showing DH’s anxiety score as measured by the GAD-7. *Note*. The solid black line represents the start of the ‘moderate’ generalised anxiety symptoms range (≥10). The horizontal orange dashed line indicates the mean score of symptoms for the baseline period. The vertical red-dotted line marks the end of baseline period and commencement of intervention.

Symptoms of generalised anxiety were measured at three time-points during the assessment phase to establish a baseline score (mean 18.3). A declining trend was seen throughout treatment. This represented ‘reliable recovery’ as DH moved from clinical to sub-clinical symptoms (≤10) *and* a change of more than four points was seen (IAPT, 2014). Figure 3 displays results for the GAD-7.

**Figure 4.**
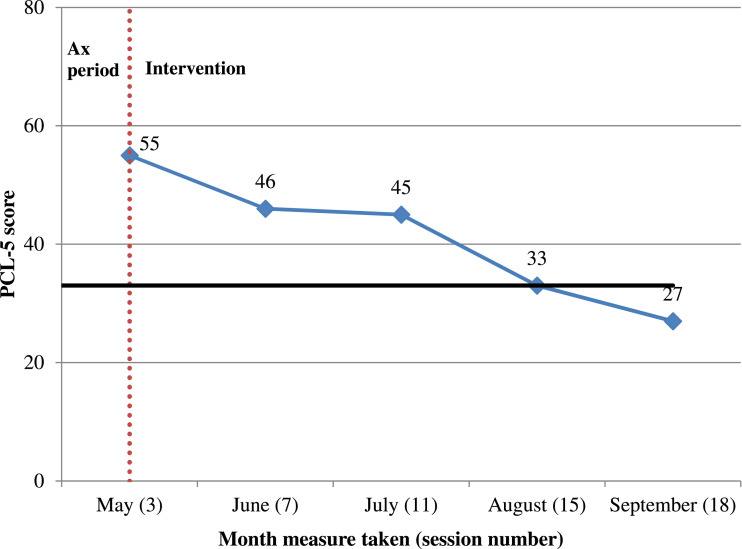
Line graph of DH’s symptoms of post-traumatic stress disorder as measured by the PCL-5. *Note.* ‘Ax period’ = Assessment period. The solid black line indicates the threshold for 'probable PTSD'. The vertical red-dotted line indicates the end of the assessment period and commencement of intervention. Only one measurement using the PCL-5 was possible during the assessment phase (see Limitations).

The PCL-5 was used monthly throughout treatment. Figure 4 shows symptoms falling from 55 in the assessment period to 27 at the end. This reflects clinically significant change since ≤33 is considered the cut-off for ‘probable’ PTSD. It also constitutes reliable change, considered to be a five to ten point fall on the scale (Weathers et al., 2013). This combination means that DH achieved ‘reliable recovery’ on this measure (IAPT, 2021).

**Figure 5.**
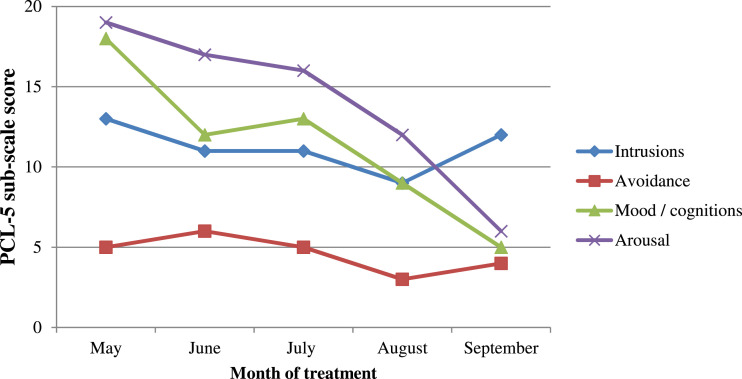
Line graph showing symptoms of post-traumatic stress disorder by PCL-5 sub-scale. *Note.* The sub-scales correspond to the four symptom clusters of post-traumatic stress disorder outlined in DSM-5.

The four subscales of the PCL-5 are shown in Figure 5. The graph reflects the challenging episode of re-experiencing while on holiday, accounting for the rise on the ‘Intrusions’ sub-scale in September. However, the ‘arousal’ and ‘mood/cognitions’ sub-scales showed notable week-on-week declines.

### Idiosyncratic Measures

DH’s score on the idiosyncratic measure ‘Ability to complete a supermarket shop, with daughter, tomorrow, on a 0-10 scale’ rose from 0 during assessment to 7 at the final session. However, DH caveated this progress by saying that this number was predicated on a situation in which all shoppers would be wearing surgical-style masks. She was much less clear on her ability to cope with masks made of black material. It was agreed that this was because they bore closer resemblance to the face-covering worn during her trauma memory.

### Outcomes by Goals. ‘In 2 Months’ Time, be Able to Walk my Smaller Dog for Short-Periods of Time With my Daughter’

DH did not achieve this goal however this was due to physical rather than mental health. Her ability to walk her dogs was compromised by mobility problems over and above psychological ones.

### In 3 Months’ Time, be Able to go Food Shopping With Daughter at Large Supermarket

DH achieved this goal around session 13. Safety behaviours were prominent but crucially she did not escape and completed the shopping.

### After 5 Months of Therapy, go on a Week-Long Holiday That Sister Booked to a Caravan Park

This goal was achieved. However, a day-trip to another town, where many people were wearing masks, resulted in flashbacks.

### Additional Outcomes

In the last few weeks of therapy, DH attended two birthday parties. At the first, she was able to order drinks from bar-staff wearing surgical masks without incident (no intrusions or hyper-arousal). During our penultimate session, DH allowed a delivery-man wearing a surgical mask into her home. Again, this passed without intrusions or hyper-arousal.

## 8 Complicating Factors

DH’s denial of the existence of COVID-19 was a complicating factor. She did not believe the virus existed and aligned herself with various conspiracy theories about the pandemic. While side-steps to such debates were made during sessions, they pertain to the progress of treatment because they fuelled confusion, hostility and incredulity toward mask-wearers. To paraphrase some of her thinking, if COVID-19 was not real, why would anyone wear a mask? And why would they do what they were told by a deceiving government? Her disbelief fostered a sense of angered confusion which prevented her from building a more coherent narrative about what was going on and why it was so difficult for her personally. Covid-denial meant that she was pre-emptively angered by mask-wearers, which likely contributed to hyper-arousal when out. One of her frequently cited pieces of evidence for COVID-19’s non-existence was neither her, nor her daughter, having become unwell. It was ironic that DH’s fear of masks had been so severe that she had stayed at home, thereby reducing the likelihood of contracting the virus, one of the few events that could plausibly have been predicted to change her mind about its veracity.

Alliance was strained by DH’s expression of overtly racist views, suggesting she did not recognise the practioner’s mixed heritage. While the severity of DH’s avoidant trauma symptoms spoke to her present, intense, fear of masks, her views about the threats posed by Muslims (particularly those with ‘backpacks’) and Black people living in an area of the city well-known for its Jamaican heritage, were important in understanding that she was psychologically pre-disposed to development of (delayed-onset) PTSD. The prevailing emotional experience of fear was not new; she had been scared of the world and what other people might do to her prior to the (scarcely believable) re-emergence of the trigger for her traumatic memories (everyone wearing masks). Given this backdrop, her reaction to the advent of mask-wearing in order to prevent the transmission of a virus she did not believe existed appeared to confirm her vision of an unjust and frightening world (in which she had also lost her youngest son to suicide).

Given the strength of these beliefs, to keep treatment focused, and in the interest of maintaining alliance, her opinions on both COVID-19’s existence and the meaning of ethnicity and race were not challenged (e.g. ‘I agree that mask-wearing is unpleasant and looks funny’ was used as a way to come alongside her distress while also side-stepping ideological debate). The practioner also held in mind DH’s age and cultural background, which meant she would have been less exposed to ethnic diversity. Further, she would have experienced fewer direct challenges to any non-acceptance of racial difference growing-up in a predominantly white-British area.

## 9 Access and Barriers to Care

The central adaptation made to the model was to accommodate DH’s physical health and mobility problems by working with her at home. While the work would not have been possible without this, it simultaneously proved a barrier. Pets served as distractions and/or safety behaviours during in-vivo exposures and reliving.

DH’s physical mobility problems gave rise to a second adaptation; emphasis on developing in-vivo exposures that were plausible in her front room (see Table 1), in favour of tasks such as site-visits that are often part of cognitive therapy for PTSD (Ehlers & Clark, 2000).

Other adaptations were not related to physical health but materials were presented in accessible language. These were more reflective of meeting literacy and comprehension needs given DH’s education level and lack of ‘psychological-mindedness’ (Bateman & Fonagy, 2006).

## 10 Follow-Up

Unfortunately, one of the limitations of this case report is that due to the nature of the therapist’s training course, longer-term follow-up was not possible (therapist left the service). DH remained under the care of the Community Mental Health team but was discharged from psychological input following this course of treatment.

## 11 Treatment Implications of the Case

On two of the three outcome measures used, the GAD-7 and PCL-5, DH achieved ‘reliable recovery’. For the former, this was by a score in the ‘minimal anxiety’ range at the end of therapy (i.e. ≤4), plus a change of more than four points during treatment. For the latter, this constituted a final score ≤33 and a >10 point decrease. On the measure of low mood, the PHQ-9, only reliable rather than clinically significant change was indicated. DH scored moderately on this at the end of treatment while also reporting a > 6 point shift overall.

The results reflect an intervention which targeted the cognitive sequelae of trauma but with no specific attention on difficulties with low mood. Nonetheless, the reliable change in this area indicates an impact of the trauma–focussed cognitive-behavioural interventions on co-morbid depressive difficulties. This is further evidenced by results on the PCL-5 ‘mood/cognitions’ sub-scale which fell from 18/28 to 5/28.

Cognitive therapy for PTSD strongly emphasises the idea that appraisals of the trauma and its sequelae contribute to distress. DH appraised intrusive thoughts in response to masks as a sign of impending and certain threat (physical harm), as they had been during her marriage. While multiple cognitive-behavioural tools were used, the reappraisal of these intrusive thoughts as serving an (overly) protective function that did not need to be suppressed but rather engaged with, was central. This provided an alternative to ineffectual thought suppression. Learning that she could be safe around masks and that mask-wearers were not desirous to hurt her was evidenced via in-vivo exposure, both during and between sessions. They taught DH that she could develop psychological skills to tolerate the arousal on contact with masks, challenged her negative predictions about the intentions of mask-wearers toward her, and finally that she could be safe without ‘sussing out’ (i.e. mind-reading) peoples’ intentions toward her. The experimental design lends credence to the assertion that symptom reduction was a result of the interventions described; internal validity was increased via regular measurement indicating stable declines in symptomology.

### Limitations

There are limitations to the present case report which affect its implications for home-based treatment of PTSD. Further to the lack of a PCL-5 measure during the baseline period, not utilising a measure of thought suppression leaves the claim that this was a key mechanism of change based on clinical judgement alone.

Stopping therapy prior to further amelioration of low mood was a further limitation, on top of discrepancy with regard to the impact of these on functioning. The PCL-5 ‘mood/cognition’ subscale showed a drop from 18 (out of 28) to 5 while DH remained in the ‘moderate’ range on the PHQ-9 (11 of 27). One of the reasons for this is that each week, DH scored maximally (i.e. 3) on the PHQ-9 item ‘thoughts of being better off dead or hurting yourself in some way’. The PCL-5 does not include an item about suicidality. DH was not acutely suicidal but believed she would be better off dead, allowing her to reunite with her youngest son. Working on such depressive cognitions, as well as the earlier identified ‘I’ll always be depressed’, would have been possible were the practioner not leaving their post, and this may have resolved the discrepancy between the two measures and allow for a more coherent conclusion in this area of the treatment’s efficacy. DH may also have benefited from some focused bereavement work, a recommendation made to members of her community care team remaining involved in the monitoring of her well-being. The obligation to end the work was disappointing, especially given that these were dictated by changes at the service-level (as opposed to client-need) and further that the results described speak to DH’s capacity and motivation for change.

## 12 Recommendations to Clinicians and Students

This case report attempts to highlight some of the hidden costs of the COVID-19 pandemic and its disproportionate impact on more vulnerable people. DH’s difficulties speak to the unpredictable implications of societal changes over the last 2 years, especially those whose psychological and physical health leaves them less visible. The central adaptation of home-based treatment, despite the facilitation of certain safety behaviours, provided efficacious therapy to someone who would have been unable to access it in its more traditional guises. Outcomes also evidence the possibility of effective home–based cognitive-behavioural treatment for (delayed-onset) PTSD in the context of significant long-term physical impairments. This is a way health-services can increase accessibility to those facing physical health related barriers to attending traditional therapy-settings, while still delivering evidence-based interventions.

This case report has described the use of home-based cognitive therapy for PTSD using a single-case experimental design. The ‘AB’ design-limitations are acknowledged but results indicate that reducing thought suppression and better contextualising of SAMs and their sequelae using stimulus-discrimination and reliving exercises, bolstered by in-vivo exposure to learn more accurately the threat posed by feared-stimuli, facilitated change.

A final recommendation is borne of a missed opportunity in the present case; to include family members/carers as co-therapists where possible during trauma-informed CBT. This is likely to enhance treatment itself as well as the longevity of any benefits accrued.

## Appendix A

Blank stimulus-discrimination worksheet completed with DH

Adapated from psychologytools.com ‘Stimulus Discrimination’ worksheet (Psychology Tools, 2022).
